# Comprehensive Insights into Mechanisms for Ventricular Remodeling in Right Heart Failure

**DOI:** 10.31083/j.rcm2512426

**Published:** 2024-11-29

**Authors:** Dongmei Jiang, Jie Wang, Rui Wang, Yun Wu

**Affiliations:** ^1^Department of General Medicine, First Affiliated Hospital of Xinjiang Medical University, 830011 Urumchi, Xinjiang, China; ^2^Department of Pharmacy, First Affiliated Hospital of Xinjiang Medical University, 830011 Urumchi, Xinjiang, China

**Keywords:** right heart failure, ventricular remodeling, autonomic nervous system, cytokines, extracellular matrix

## Abstract

Ventricular remodeling in right heart failure is a complex pathological process involving interactions between multiple mechanisms. Overactivation of the neuro-hormonal pathways, activation of the oxidative stress response, expression of cytokines, apoptosis of cardiomyocytes, and alterations of the extracellular matrix (ECM) are among the major mechanisms involved in the development of ventricular remodeling in right heart failure. These mechanisms are involved in ventricular remodeling, such as myocardial hypertrophy and fibrosis, leading to the deterioration of myocardial systolic and diastolic function. A deeper understanding of these mechanisms can help develop more effective therapeutic strategies in patients with right heart failure (RHF) to improve patient survival and quality of life. Despite the importance of ventricular remodeling in RHF, there are a limited number of studies in this field. This article explores in-depth historical and current information about the specific mechanisms in ventricular remodeling in RHF, providing a theoretical rationale for recognizing its importance in health and disease.

## 1. Introduction

Right heart failure (RHF) [1] is one of the most serious outcomes of pulmonary hypertension (PAH) and leads to ventricular remodeling, which in turn affects the normal function of the heart. The free wall of the right ventricle (RV) is thinner than that of the left ventricle (LV), which is usually between 3 and 5 millimeters thick in adults, and the RV is about one-third to one-sixth smaller in mass than the LV. Cardiomyocytes in the RV are about 15% smaller than those in the LV; however, the collagen content of the RV is increased, by about 30% [2]. Ventricular remodeling is a complex biological process involving the interaction of multiple mechanisms. Myocardial remodeling is a broad definition for any change in the heart’s structure and function, which can be categorized into physiological and pathological types. Physiological myocardial remodeling is a beneficial adaptive process, which results in decreased wall stress, increased cardiac pump function, and improved vascularization. Early pathologic myocardial remodeling serves to reduce ventricular wall stress and temporarily protect cardiac pump function but eventually progresses to heart failure and death. Pathological myocardial remodeling is associated with cellular hypertrophy, rhabdomyolysis, glycogen accumulation, chronic oxidative stress, cell death, inflammation, fibrosis, decreased capillary density, and electrical disturbances [3]. Over-activation of the neuro-hormonal pathways, oxidative stress, cytokine expression, cardiomyocyte apoptosis, and extracellular matrix (ECM) alterations are the main pathophysiological mechanisms that comprise ventricular remodeling in RHF.

**Fig. 1.**
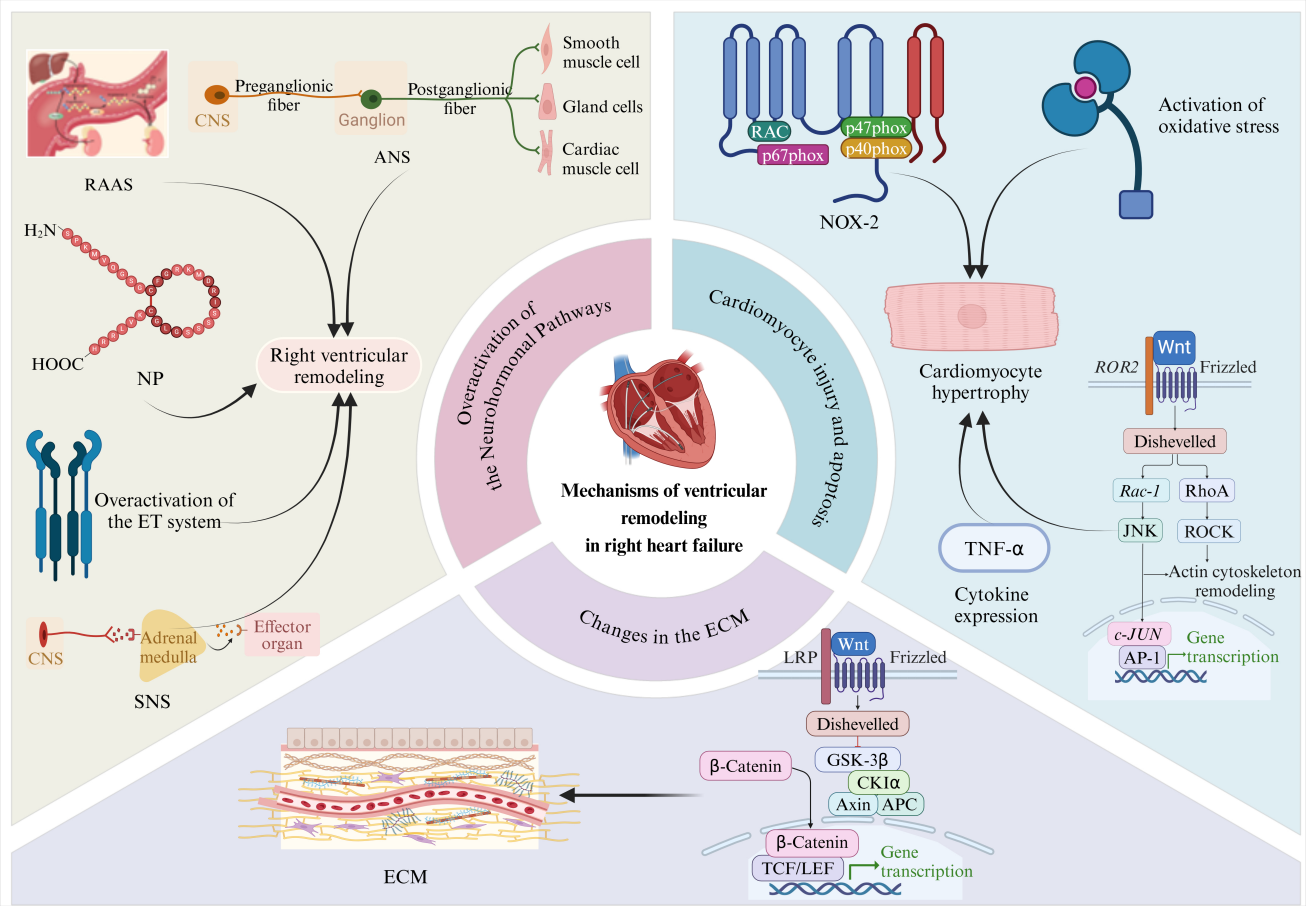
**Mechanisms associated with ventricular remodeling in right heart failure**. The upper left panel shows the effect of neuro-hormonal pathway activation on ventricular remodeling in patients with right heart failure. The upper right panel highlights the effects of cardiomyocyte injury and apoptosis on right ventricular remodeling. The lower part of this figure shows the effect of extracellular matrix alterations on right ventricular remodeling. Overactivation of the neuro-hormonal pathways: RAAS, renin-angiotensin-aldosterone system; ANS, autonomic nervous system; NP, natriuretic peptide; ET, endothelin; SNS, sympathoadrenal system. Cardiomyocyte injury and apoptosis: TNF-α, tumor necrosis factor-alpha; ECM, extracellular matrix; CNS, central nervous system; RAC, guanosine triphosphate (GTP)-binding protein Rac; JNK, *c-JUN* (c-jun proto-oncogene) N-terminal kinase; ROCK, rho-associated protein kinase; LRP, low-density lipoprotein (LDL) receptor-related protein; GSK-3β, glycogen synthase kinase-3β; CKIα, casein kinase I α; TCF, T-cell factor; LEF, lymphoid enhancer factor; AP-1, activator protein-1; ROR2, receptor tyrosine kinase-like orphan receptor 2; NOX-2, nicotinamide adenine dinucleotide phosphate oxidase-2; Rac-1, ras-related C3 botulinum toxin substrate 1; RhoA, ras homologous gene family member A; APC, adenomatous polyposis coli.

Hyperactivation of the neuro-hormonal pathways play a crucial role in the development and progression of RHF. Overactivation of the renin-angiotensin-aldosterone system (RAAS) [4], the sympathetic nervous system, and neuro-hormonal pathways, such as natriuretic peptides, are collectively involved in the complex process of ventricular remodeling, such as myocardial hypertrophy, cardiomyocyte apoptosis, and fibrosis. In addition, overactivation of neuro-hormonal pathways causes vasoconstriction and sodium retention, further decreasing cardiac function. During ventricular remodeling, increased expression of several cytokines such as tumor necrosis factor-alpha (TNF-α), connective tissue growth factor (CTGF), and endothelin (ET) leads to apoptosis and fibrosis of cardiomyocytes, which in turn promotes ventricular remodeling. In RHF, the rate of apoptosis of cardiomyocytes increases, and the number of cardiomyocytes decreases, which affects both myocardial systolic and diastolic function. Increased expression of these cytokines leads to apoptosis and fibrosis of cardiomyocytes, which accentuates ventricular remodeling. ECM alterations are also an essential mechanism of ventricular remodeling in RHF. In RHF, myocardial ECM alterations include an imbalance in the proliferation and degradation of collagen fibers, leading to altered alignment of cardiomyocytes and interstitial fibrosis. This ECM alteration further affects myocardial diastolic function and promotes the development of ventricular remodeling (Fig. 1).

In summary, the mechanisms responsible for ventricular remodeling in RHF include overactivation of the Neuro-hormonal Pathways, activation of oxidative stress, expression of cytokines, apoptosis of cardiomyocytes, and alterations in the ECM. There is a close interaction between these mechanisms involved in the complex biological process of ventricular remodeling in RHF. Therapeutic approaches that target these key mechanisms can help to significantly improve the clinical symptoms of RHF and effectively slow the progression of ventricular remodeling. A deeper understanding of these mechanisms will provide important guidance for developing more effective therapeutic strategies, to increase survival and improve the quality of life of patients with RHF.

## 2. Overactivation of the Neuro-hormonal Pathways

### 2.1 The Autonomic Nervous System

The autonomic nervous system (ANS), also known as the vegetative nervous system or the involuntary nervous system, is an important part of the peripheral nervous system of vertebrates. Through the differentiation and development of the somatic nerves, a functionally independent nervous system is formed, which is responsible for regulating the internal environmental homeostasis of the body and the function of organs. The ANS and biologically active substances such as vasopressin, angiotensin, oxytocin, and cytokines play a synergistic role in the functional regulation of the cardiovascular system, working together to maintain the stability of the body’s internal environment and the ability to adapt to changes in the external environment. However, in pathological states such as heart failure and hypertension, the interactions between the ANS and these regulatory factors change significantly, interfering with the normal regulatory mechanisms of the cardiovascular system and leading to a series of imbalances in physiological and pathological processes [5].

The ANS, named after its function in regulating the activity of internal organs, vascular smooth muscle, cardiac muscle, and glands, can autonomously regulate the activity of these tissues and organs, ensuring the stability of the internal environment of the organism and adapting to changes in the external environment. The ANS is mainly comprised of three core components: the ANS, the parasympathetic nervous system, and the enteric nervous system. The scope of this paper is limited to the sympathetic and parasympathetic nervous systems to investigate their functions and regulatory mechanisms in physiological and pathological states. They synergistically regulate the activity and secretion of the body’s organs, blood vessels, smooth muscles, and glands [6]. Recently, study has shown that the regulation of cardiac neuromodulation extends beyond the central nervous system’s external influence to include a specialized, intrinsic cardiac nervous system (ICNS) [7]. This sophisticated network comprises sensory neurons, both pre-and postganglionic parasympathetic neurons, as well as biphenotypic neurons capable of releasing neurotransmitters of the sympathetic and parasympathetic systems. Together, these elements orchestrate a complex neural network crucial for the nuanced regulation of the cardiovascular system [8].

The ANS plays a pivotal role in regulating cardiac function. Specifically, it is responsible for precisely and efficiently regulating myocardial contractile force, blood pressure, and heart rhythm. Cardiac sympathetic preganglionic fibers originate from thoracic segments 1 to 6 of the spinal cord and reach myocardial tissues and coronary vessels mainly through postganglionic fibers emitted by stellate ganglionic exchange neurons. Postganglionic neurons contain the enzyme tyrosine hydroxylase to synthesize norepinephrine (NE), the main neurotransmitter of sympathetic nerves. Sympathetic activation in the body is characterized by increased circulating blood concentrations of catecholamines (CA), including amines such as NE, epinephrine, and dopamine [9]. The parasympathetic preganglionic fibers of the heart emanate from the dorsal nuclei of the vagus nerve in the medulla oblongata. After exchanging neurons in the cardiac ganglionic plexus, they innervate primarily the atria and the cardiac conduction system. Parasympathetic nerves, in turn, act to slow the heart rate and reduce myocardial contractility by releasing acetylcholine. However, there are no reports of altered parasympathetic tone in RHF in the current literature, which should receive further attention in future studies. The critical role of the ANS in modulating the ST-segment elevation phenomenon and tachyarrhythmic events in patients with Brugada syndrome has been revealed [10]. The sympathetic nervous and parasympathetic systems function antagonistically, synergistically, or independently of each other to maintain the functional balance of the autonomic effector organs and to ensure the homeostasis of the organism’s internal environment and its adaptation to changes in the external environment. Several studies have confirmed that imbalances in the ANS, that is, abnormal increases or decreases in sympathetic or parasympathetic tone, are closely associated with the onset and progression of a variety of diseases, such as arrhythmias after myocardial infarction [11], Obstructive sleep apnea [12], diabetes [13] and Parkinson’s disease [14]. Carotid pressure receptor stimulation (CBS) is a non-pharmacological autonomic modulation intervention. By analyzing the effects of CBS on wild pachydermine-induced PAH and its underlying mechanisms, it was suggested that CBS could significantly attenuate right ventricular dysfunction, improve pulmonary artery and right ventricular remodeling, and thus improve the survival rate of rats with monocrotaline-induced PAH [15]. The ANS, which influences myocardial contractility, blood pressure, and heart rhythm, is essential for controlling cardiac function. Numerous diseases are strongly linked to their imbalance, and comprehending the disease process requires a thorough grasp of the autonomic nerve system’s regulatory processes.

### 2.2 The RAAS

RAAS [4] is an important blood pressure regulatory system in the body, consisting mainly of renin, angiotensin, and aldosterone. Renin is a protein hydrolyzing enzyme secreted by the periglomerular cells of the glomerulus, and when it enters the circulation, it interacts with α2 globulin produced by the liver, which in turn produces angiotensin I (Ang I), a decapeptide substance. Renin secretion is complexly regulated by multiple mechanisms, including stimulation of renal pressure receptors, changes in distal tubular ion transport, β-1adrenergic receptor (AR)-mediated sympathoexcitatory effects, and a negative feedback mechanism for angiotensin II (Ang Ⅱ). Renin may catalyze the conversion of angiotensinogen to Ang I once it is activated. The circulatory system and a local angiotensin-converting enzyme hydrolyze the Ang I to produce the physiologically active Ang Ⅱ [16]. Enzymatic conversion in the pulmonary system transforms Ang I into Ang Ⅱ and III. Ang Ⅱ exerts a potent vasoconstrictive effect and prompts the adrenal medulla to secrete adrenaline. In turn, it stimulates sympathetic nerve terminals to release norepinephrine, collectively contributing to an antihypertensive response. Moreover, Ang Ⅱ and III both trigger the adrenal cortex to secrete aldosterone, a pivotal hormone in water and sodium retention, raising blood pressure. Conversely, Ang Ⅱ receptors play a vasodilatory role, countering remodeling through anti-proliferative and anti-apoptotic actions. Within the kidneys, Ang Ⅱ receptors are implicated in modulating sodium reabsorption in the proximal tubules, affecting prostaglandin synthesis and secretion, and enhancing the sodium reduction response to increased renal perfusion pressure [17].

In the heart, the RAAS regulates cardiac function, modulates coronary artery resistance, and inhibits cardiomyocyte growth. It also significantly affects vascular structure and the operation of the coagulation system, and it can control vasodilation and contraction. The body’s ability to maintain blood pressure homeostasis and heart function depends heavily on the RAAS. Diseases, including hypertension and cardiac hypertrophy, can arise when the RAAS is not working properly. Studies have confirmed that the Janus kinase 2 (*JAK2*)/signal transduction and activation of transcription 3 (STAT3) signaling pathway plays a key role in regulating the expression of transforming growth factor II, collagen type I alpha 1 (COL1A1), and myosin heavy chain 7 (Myh7), which in turn participates in Ang II-induced myocardial hypertrophy and fibrosis, and ultimately promotes the development of cardiac remodeling [18]. Dehydroepiandrosterone effectively promotes the improvement of pulmonary hemodynamics, alleviates the process of pulmonary vascular remodeling, and enhances cardiac function through the inhibition of the STAT3 signaling pathway, thereby attenuating the remodeling phenomenon of the RV [19]. Given the limitations of the scope of this study, clinical trials have not been able to adequately confirm the effectiveness of RAAS inhibition therapy in patients with coronary artery disease and RHF [20]. To prevent and treat RHF, a thorough understanding of the physiology and pathological mechanisms underlying the RAAS is essential. By revealing the association between its regulatory mechanisms and the development of diseases, it is expected to provide new ideas and methods for clinical treatment, thereby improving patients’ quality of life and reducing the disease burden.

### 2.3 Natriuretic Peptide

Natriuretic peptide (NP) [21] also maintains homeostasis within the cardiovascular system, with potent natriuretic, diuretic, vasodilator, and inhibitory effects on sympathetic nerve activity. There are five main types of natriuretic peptides: atrial natriuretic peptide (ANP), brain natriuretic peptide (BNP), C-type natriuretic peptide (CNP), dendroaspis natriuretic peptide (DNP), and uroguanosine [22]. ANP and BNP play a local (autocrine/paracrine) regulatory role in the heart, and another natriuretic peptide, CNP, acts as a regulator within the vascular wall. In this review, we will only discuss ANP, BNP, and CNP. Initial purification of BNP was achieved from porcine brain extracts, yet subsequent studies have shown that the heart is the primary source of circulating BNP. In addition, CNP is produced by both nerve tissue and vascular endothelial cells. The natriuretic peptide is present in ventricular septal particles and can be secreted from these particles. Its secretion depends on volume expansion and increased pressure load in the ventricles. When cardiomyocytes are stretched and stimulated, they secrete B-type natriuretic peptide protomer precursors, which are cleaved into biologically active and inactive natriuretic peptides under endonuclease. NP has several effects on the heart. First, it dilates blood vessels, causing a decrease in cardiac output and a reduction in cardiac load, with diuretic, sodium, and drainage effects. ANP and BNP exhibit classical endocrine actions in vascular smooth muscle and renal units. Second, natriuretic peptides can lower blood pressure and can also lead to a decrease in cardiac load. During overloading of cardiac function or squeezing of cardiac capacity, natriuretic peptides are activated, leading to elevated levels of natriuretic peptides, which help diagnose heart failure. There are significant associations between the levels of these two natriuretic peptides at moderate concentrations and the risk of developing structural heart disease and heart failure [23].

The three membrane-bound natriuretic peptide receptors known as natriuretic peptide receptor-A, natriuretic peptide receptor-B (NPR-B), and natriuretic peptide receptor-C are also included in the natriuretic peptide system [24]. The human natriuretic peptide type A (*NPPA*) gene, found on chromosome 1, region 1p36.21, encodes an ANP precursor longer than 2 kb and has three exons. Approximately 10 kb upstream of the *NPPA*, the natriuretic peptide precursor (*NPPB*) gene produces a BNP precursor with a similar structure; it is likely that the two genes evolved via gene duplication [25]. Brain *NPPB* and N-terminal brain natriuretic peptide precursor levels must be prioritized by healthy persons, and this is because variations in the concentrations of these markers might indicate early cardiac responses [26]. The *NPPB* gene is upregulated in response to mechanical stretching that may occur in hypertension or other risk factors for heart failure. In addition, the promoter of the *NPPB* gene contains a kinase element associated with extracellular signaling stimulated by the RAAS, which regulates gene expression and subsequent biological processes [27]. Upon binding to the NP receptor, *NPPB* can trigger a series of biological effects, including the promotion of diuretic and natriuretic processes, the lowering of blood pressure, and the attenuation of the activity of the RAAS. Together, these effects help maintain the body’s water-salt balance and cardiovascular homeostasis [28]. In the GPx3-deficient pulmonary artery banding animal model, an exacerbation of adverse RV remodeling was observed, accompanied by a significant increase in the levels of CTGF, transforming growth factor-β (TGF-β), and ANP expression in the RV [29]. Combination therapy with Ang II receptor antagonists and neprilysin inhibitors significantly reduced RV systolic blood pressure and attenuated RV hypertrophy and dilatation in PAH rats. However, similar therapeutic effects were not observed in a rat model of isolated RHF [30].

### 2.4 The Endothelin System

Over-activation of the ET system manifests as by excessive endothelin (ET) secretion, which in turn triggers excessive contraction of the cardiovascular system and elevated blood pressure. ET [31] is a 21-amino acid peptide derived from big ET by the action of a converting enzyme, and includes three isoforms of ET-1, ET-2, and ET-3. Among them, ET-1 is mainly produced by vascular ET cells, and its effects are mediated through two G protein-coupled receptors, ET-A and ET-B, which are widely distributed in various human body tissues, especially in cardiac tissues. ET-1 is potent vasoconstrictor, myocardial tone, and mitogenic effects, regulates salt and water balance, and maintains vascular tone and blood pressure stability [32]. Human cardiomyocytes also express ET. ET is mostly expressed in healthy mesenchymal and ET cells that have been isolated from the ventricles, ET expression in cardiomyocytes noticeably increases under pathological conditions such as ischemic cardiomyopathy [33]. As a multifunctional regulator, TGF-β [34] is essential for cell division, proliferation, and apoptosis. Vascular tone and cell proliferation are two physiological processes regulated by ET-1 mRNA, which is expressed in vascular ET cells in response to TGF-β. Smad protein, a crucial transcription factor in the TGF-β signaling pathway that reacts to TGF-β stimulation and controls downstream gene expression, is required for this induction [35]. During ventricular remodeling, the ET system may be overactivated due to myocardial injury and increased loading, leading to vasoconstriction and further aggravating the burden on the heart. GTP-binding proteins connect with ET-A and ET-B receptors, which pair with phospholipase C (PLC). Phosphatidylinositol is hydrolyzed during PLC activation, rapidly producing 1,4,5-trisphosphatidylinositol (IP3) and causing 1,2-diacylglycerol (DG) to accumulate over time. IP3, a crucial intracellular signaling molecule, can dramatically increase intracellular calcium ion concentration, which in turn causes a vasoconstrictor response [36]. ET-A receptors on vascular smooth muscle cells are the major pathway mediating vasoconstrictor effects, and their potency is ranked as ET-1 equivalent to ET-2, both stronger than ET-3 [37]. Furthermore, ET-B receptors are more important for controlling baseline blood pressure and vascular tone than are ET-A receptors. This role is defined by the capacity to shield the vascular system from the potent vasoconstrictive effects of endogenous ET [38]. ET-1 enhances exogenous norepinephrine-induced contractions via ET-A receptors [39]. Simultaneously, it stimulates the heart to produce atrial natriuretic peptide [40] and nitric oxide production by vascular ET cells [41]. This process may be regarded as an organismal autocrine feedback mechanism for regulating the ET-triggered vasoconstrictor response, thus maintaining homeostasis via maintaining vascular tone. In addition, ET may be involved in ventricular remodeling by promoting apoptosis and fibrosis in cardiomyocytes and influencing the heart’s electrophysiological activity. Its cardiovascular effects and pro-hypertrophic function are particularly significant in heart failure [42]. Ang II may either directly or indirectly influence ET-1 secretion during the pathologic progression of heart failure by encouraging the generation of pressors. Angiotensin-converting enzyme activity can also be increased to further boost ET-1 and vasopressor production, which may worsen the pathophysiologic alterations in heart failure [43]. ET-1 signaling is upregulated in the heart, kidneys, nervous system, and vasculature during the pathophysiology of hypertension. This results in an imbalance in the regulatory mechanisms of these organs and systems [44]. An increase in ET-1 production characterizes the pathological conditions of hypertrophic heart disease and heart failure. Prolonged administration of antagonists of the ET-A-type receptor is efficacious in ameliorating modifications in cardiac gene expression resulting from these pathological processes [45]. The intricate signaling pathways that govern the hypertrophy of cardiomyocytes remain incompletely understood; nonetheless, ET-1 stimulates the proliferation of adrenocortical zona glomerulosa cells through the induction of a tyrosine kinase-dependent mitogen-activated protein kinase (MAPK) cascade response [46]. In a rat model of right ventricular hypertrophy, right ventricular nerve growth factor (NGF) protein expression was observed to be upregulated in parallel with ET-1 mRNA expression, suggesting that the ET-1/NGF signaling pathway plays an important role in cardiac development and is involved in pathological processes in disease states [47].

### 2.5 Activation of the SNS

Activation of the sympathoadrenal system (SNS) is also one of the important mechanisms of ventricular remodeling in RHF. During activation of the SNS, the adrenal glands secrete excessive amounts of CA, including epinephrine and norepinephrine, to maintain cardiovascular systemic function [48]. Activation of this system leads to an increase in the concentration of calcium ions in cardiomyocytes and an increase in myocardial contractility, but it also leads to apoptosis and fibrosis of cardiomyocytes, which in turn aggravates ventricular remodeling. The SNS is a system of sympathetic nerves and the adrenal medulla that primarily responds to emergencies. ARs can be categorized into several subtypes, such as α1, α2, β1, β2, and β3, all of which belong to the G protein-coupled receptor superfamily. Norepinephrine primarily stimulates the α receptor and two subtypes of the β1 receptor, whereas epinephrine can activate all subtypes of the α and β-AR.

The SNS influences gene expression in cardiomyocytes primarily through the release of catecholamine neurotransmitters, which alters the structure and function of cardiomyocytes. This process involves the activation and regulation of several signaling pathways. These include the following pathways: (1) β-AR signaling pathway: The β1-AR dominates the heart, making up about 80% of the cardiac β-ARs. β2-AR makes up about 20% [49]. RHF and LHF, although similar in β-AR signaling regulation, have significantly different clinical responses to β-adrenergic blockers. In PAH-induced RHF, the β1, α1, and DA1 receptors are downregulated, cyclic adenosine monophosphate (cAMP) levels are decreased, and G protein-coupled receptor kinases-2 (GRK-2) activity is elevated, resulting in impaired positive inotropic responses [50]. A study confirmed that bisoprolol significantly inhibited the pathological process of right ventricular dilatation and effectively slowed down the rate of cardiac decompensation, which in turn delayed the progression of RHF [51]. CA bind to receptors to activate the G protein-coupled receptor (GPCR) pathway, producing second messengers such as cAMP, which in turn activate kinases such as Protein kinase A, phosphorylate transcription factors, and proteins that regulate cardiomyocyte gene expression. Upon activation of GPCR, phosphoinositide 3-kinase gamma (PI3Kγ) activates Akt/protein kinase B (PKB), triggering pathological hypertrophy [52]. Beta-blockers are currently the first line of treatment for heart failure. (2) Calcium ion signaling pathway: CA neurotransmitters can also increase the intracellular concentration of calcium ions by activating L-type calcium channels on the cardiomyocyte membrane. Calcium ions, key intracellular second messengers, are involved in regulating various cellular processes, including cardiomyocyte contraction, diastole, growth, and apoptosis. (3) The MAPK signaling pathway, which includes essential elements such as p38 MAPK and extracellular signal-regulated kinases, can be activated by CA neurotransmitters. This pathway allows for the precise control of gene expression processes in cardiomyocytes. (4) Calcineurin/nuclear factor AT (NFAT) signaling pathway: Through the activation of calcineurin, a transcription factor that controls the expression of several genes linked to cardiomyocyte shape and function, catecholamine neurotransmitters can also activate the NFAT signaling pathway [53]. In RHF, activation of the SNS is crucial for the body’s emergency reaction and homeostasis maintenance during remodeling in heart failure. Alternatively, over-activation could worsen cardiovascular instability, and result in heart disease and hypertension. Therefore, maintaining cardiovascular health depends on regulation of these diverse systems.

## 3. Cardiomyocyte Injury and Apoptosis

### 3.1 Activation of Oxidative Stress

Oxidative stress initiates a cascade of detrimental processes within cardiomyocytes, including peroxidation of membrane lipids and protein denaturation, thereby compromising cellular integrity and function. This stressor not only accelerates cardiomyocyte death but also fuels the progression of ventricular remodeling. Additionally, oxidative stress elicits an inflammatory response that exacerbates necrosis of cardiomyocytes. One key manifestation of oxidative stress in cardiomyocytes is the induction of apoptosis, a programmed cell death process mediated by proteins such as p53 and Bax. Furthermore, oxidative stress triggers an abnormal surge in intracellular Ca^2^⁺ ion levels, resulting in calcium overload which exacerbates cardiomyocyte injury. Pathological conditions such as myocardial ischemia, reperfusion, and hypertension are characterized by increased levels of oxidative stress in cardiomyocytes, primarily stemming from free radicals and mitochondrial nicotinamide adenine dinucleotide phosphate (NADPH) oxidase activity. This oxidative stress directly impairs mitochondrial function by accentuating the opening of the mitochondrial permeability transition pores, causing a decline in mitochondrial membrane potential. Consequently, mitochondrial swelling and rupture ensue, releasing cytochrome C (cyt-C) into the cytosol. This event triggers the caspase cascade, ultimately initiating apoptosis in cardiomyocytes. The sthdy in apoptosis signal-regulating kinase 1 (ASK1) gene-deficient mice confirmed the critical role of the reactive oxygen species (ROS)/ASK1 pathway in necrotic and apoptotic cell death, providing important clues to ASK1 as a potential therapeutic target for reducing left ventricular remodeling after myocardial infarction [54]. Inhibition of matrix metalloproteinases (MMPs) induction and myocardial remodeling by TNF-α blocking proteins in a dog pacing-induced heart failure model confirmed their ability to block local matrix metalloproteinase production in cardiac TNF-α overexpressing mice [55].

In summary, activation of oxidative stress has significant negative effects on the myocardium, leading to myocardial injury and ventricular remodeling. Therefore, therapeutic strategies targeting oxidative stress are important for improving the symptoms of RHF. Oxidative stress damages cellular macromolecules, including many key cardiac myosin proteins, membrane lipids, especially cardiolipin, and mitochondrial DNA (mtDNA), ultimately leading to cardiomyocyte death and even myocardial remodeling and dysfunction. A study by Fang *et al*. [56], bromodomain-containing protein 4 (BRD4) knockdown dramatically increased the production of nuclear factor erythroid 2-related factor-2 (Nrf2) and heme oxygenase 1 (HO-1) proteins while suppressing TLR4 and nuclear factor-kappa B (NF-κB) phosphorylation. Subsequent research also revealed that BRD4 silencing decreased inflammatory cytokines, attenuated oxidative stress, and decreased ventricular hypertrophy; however, all these effects were markedly inhibited in Toll-like receptor 4 (TLR4) overexpression. Bogaard *et al*. [57] showed that the degree of fibrosis in a hypoxia model was synchronized with the reduction of Nrf2 and HO-1 expression and restoration of Nrf2, and that HO-1 signaling prevented adverse right ventricular remodeling and protected RV function.

### 3.2 Cytokine Expression

Various cytokines such as TNF-α, CTGF, and ET are involved in ventricular remodeling in RHF. The expression of TNF-α is increased in RHF. Increased expression of these cytokines leads to apoptosis and fibrosis of cardiomyocytes, which aggravates ventricular remodeling. The specific mechanisms of action of these cytokines are as follows: The expression level of TNF-α is increased in the state of RHF. It can induce apoptosis and necrosis of cardiomyocytes by binding to receptors on the surface of cardiomyocytes, further promoting the progression of ventricular remodeling. In addition, TNF-α can mediate the inflammatory response, which can cause infiltration and aggregation of inflammatory cells and exacerbate myocardial injury. Apart from its detrimental inotropic effects, TNF-α is essential for ventricular remodeling. Following cardiac stress, TNF-α, transforming growth factor β, and pro-inflammatory cytokines such as interleukin (IL)-1, -12, -8, and -18 are often involved as mediators [58]. Recombinant mouse IL-17 significantly exacerbated cardiomyocyte hypertrophy and apoptosis in hypoxia. In *in vitro* experiments, IL-17 inhibited cardiomyocyte viability and triggered the apoptotic process of cardiomyocytes through the STAT3 signaling pathway, exacerbating right ventricular remodeling, regardless of whether they were under normoxic or hypoxic conditions [59]. Magnolol effectively alleviated the hypoxia-induced hypertrophy and fibrosis process in the RV of PAH rats and inhibited right ventricular remodeling by blocking the *JAK2*/STAT3 signaling pathway [60]. In rat lung tissues treated with celastrol, there was a significant reduction in macrophage infiltration, down-regulation of pro-inflammatory cytokine expression levels, up-regulation of anti-inflammatory cytokine expression levels, and effective inhibition of the activation of the NF-κB signaling pathway, and ultimately attenuation of right ventricular remodeling [61]. *In vitro* experiments have shown that poly (adenosine diphosphate ribose) polymerase 1 (PARP1) overactivation exacerbates cardiomyocyte dysfunction through upregulation of pyruvate kinase muscle isoform 2 expression and its nuclear function, which in turn promotes glycolytic gene expression as well as the activation of NF-κB-dependent proinflammatory factors, which together exacerbate cardiomyocyte dysfunction. Pharmacological or genetic inhibition targeting PARP1 or forced pyruvate kinase M2 (PKM2) tetramerization are effective in mitigating maladaptive remodeling of cardiomyocytes, thereby improving RV function [62]. The CTGF, a member of the cellular communication network (CCN) family, is a stromal cell protein composed of six homologous cysteine structural domains [63]. In a PAH rat model of cardiac fibroblasts, microRNA-1 (miR-1) antagonist transfection significantly reduced the expression of collagen I, collagen III, α-smooth muscle actin, and CTGF, and inhibited the enhancement of phosphorylated PI3K and phosphorylated Akt. This result reveals that miR-1 inhibition alleviates right ventricular hypertrophy and fibrosis in PAH rat models, and that the underlying mechanism involves the regulation of the PI3K/Akt signaling pathway [64]. The CTGF plays an important role in remodeling the ECM in the myocardium. It stimulates fibroblast growth and collagen synthesis, leading to abnormal deposition of myocardial ECM, further affecting myocardial diastolic function. The CTGF also induces hypertrophy and apoptosis of cardiomyocytes and participates in the ventricular remodeling process. Li *et al*. [65] found that the use of vascular peroxidase 1 (VPO1) siRNA inhibited hypochlorous acid (HOCl) production, extracellular signal-regulated kinase phosphorylation, and cardiac hypertrophy. The VPO1 enzyme catalyzed the production of HOCl, which in turn facilitated the process of RV remodeling in a rat model of PAH, a process that led to activation of the extracellular regulated kinase (ERK) signaling pathway. ET is a potent vasoconstrictor that promotes cardiac contraction and cardiac diastole. In the state of RHF, the expression level of ET increases and causes hypertrophy and apoptosis of cardiomyocytes by binding to its receptor. In addition, ET can stimulate the release of calcium ions from cardiomyocytes, leading to enhanced contraction of cardiomyocytes, but long-term, these effects can cause damage and necrosis of cardiomyocytes. In summary, various cytokines are involved in ventricular remodeling in RHF, inducing apoptosis and necrosis of cardiomyocytes and promoting the progression of ventricular remodeling through different mechanisms. Therapeutic strategies targeting these cytokines may provide new therapies for improving the symptoms of RHF.

### 3.3 Cardiomyocyte Apoptosis

Apoptosis of cardiomyocytes is one of the important mechanisms of ventricular remodeling in RHF. Cardiomyocyte apoptosis is the active initiation and execution of a series of ordered biochemical reactions in cardiomyocytes in response to various stimuli, leading to programmed cell death. This process leads to a decrease in the number of cardiomyocytes, which in turn triggers the destruction of myocardial tissue. Cardiomyocyte apoptosis in the peri-infarct and remote infarct zones is closely associated with ventricular dilatation and reduced ventricular systolic function after myocardial infarction, with the correlation appearing to be more pronounced in the remote infarct zone. The prolonged presence of apoptosis in the noninfarcted area allows continued death of surviving cardiomyocytes, which limits structural and functional recovery after infarction and promotes the development of ventricular remodeling. The remodeling process also involves sliding myofibers of nonischemic myocardium against each other, causing thinning of the ventricular wall and enlargement of the ventricular cavity. This compensatory response results in an increase in stroke volume and a reduction in the extent of peripheral myocardial shortening. During inter-cellular sliding, this structural rearrangement of myofiber components requires the death of individual cardiomyocytes to cause a change in position between the cells. In addition, apoptosis in the peri-infarct zone and cardiomyocytes in the remote infarct zone may play an important role in acute and chronic ventricular wall remodeling through these mechanisms. If the area of the myocardium in which hibernation occurs is large, neuroendocrine mechanisms may be triggered to initiate or accelerate the process of cardiac remodeling, resulting in an undesirable vicious cycle. Magnesium lithospermate B administration inhibited cardiomyocyte hypertrophy and decreased the expression of NADPH oxidase (NOX) (especially NOX2 and NOX4) and VPO1, which effectively prevented hypoxia-induced RV remodeling process in PAH rats by blocking the activation of NOX/VPO1 and ERK signaling pathways [66]. Mechanistic study demonstrated that the use of betaine significantly increased the expression of Rho A, Rho-associated protein kinase 1 (ROCK1), and ROCK2, which alleviated pulmonary vascular remodeling and cardiomyocyte apoptosis through the altered Rho A/ROCK signaling pathway, ultimately attenuating RHF [67].

Thus, apoptosis of cardiomyocytes is one of the important mechanisms of ventricular remodeling and profoundly impacts its development and the recovery of cardiac function. Strategies to prevent and treat cardiomyocyte apoptosis may provide new therapies to improve ventricular remodeling and cardiac function.

## 4. Changes in the ECM

Another key process in RHF is the change in ECM. The ECM is responsible for mechanical and electrical connections and serves as the structural foundation of the heart. Excessive ECM buildup in heart failure increases ventricular stiffness and interferes with electrical coupling, resulting in alterations in conduction, arrhythmias, and sudden death [67]. Alterations in the ECM include an imbalance in the proliferation and degradation of collagen fibers, leading to the disorganization of cardiomyocytes and interstitial fibrosis, which further affects the diastolic and contractile functions of the myocardium. Myocardial ECM is mainly composed of collagen, matrix metalloproteinases, and cell surface adhesion molecules, and plays a key role in maintaining cardiac shape and transmitting intercellular signals. During ventricular remodeling, the balance between synthesis and degradation of the cardiac ECM is disrupted. Excessive deposition of collagen leads to stiffening of the myocardial ECM and affects the diastolic function of the myocardium. In contrast, the activity of matrix metalloproteinases (MMPs) is finely regulated, and their activity status directly influences the degradation process of the ECM in the myocardium and plays an important role in ventricular remodeling. Furthermore, through controlling the expression of MMPs and tissue inhibitory factors, a range of growth factors and cytokines, including TGF-β and TNF-α, significantly impact the equilibrium of synthesis and degradation of the cardiac ECM. During ventricular remodeling, the expression levels of these factors are significantly altered, further regulating the remodeling process of the myocardial ECM. Alterations in the cardiomyocyte ECM affect the morphology and function of cardiomyocytes involved in the processes of cardiomyocyte growth, differentiation, and apoptosis. In an experimental model of PAH, prophylactic application of ACTRIIA-Fc significantly optimized hemodynamic parameters, inhibited RV hypertrophy, improved RV function, and alleviated the phenomenon of small artery remodeling [68]. By using LGK-974, a Wnt signaling inhibitor, targeting and regulating the Wnt/β-catenin and FOS-like (FOSL) signaling axes, RV function was significantly improved, which in turn suppressed the pathological remodeling genes of the RV [69]. ECM remodeling is a key mechanism in RHF, which involves an imbalance between collagen fiber proliferation and degradation, leading to disturbed cardiomyocyte arrangement and interstitial fibrosis, and affecting myocardial diastolic function. The ECM consists of collagen, MMPs, and other proteins, and the balance between their synthesis and degradation is crucial in ventricular remodeling. Growth factors and cytokines regulate this process, and ECM alterations not only affect myocardial morphology and function, but also participate in cell growth, differentiation, and apoptosis. Therefore, therapeutic strategies targeting ECM are important for improving ventricular remodeling and cardiac function.

## 5. Conclusions

RHF ventricular remodeling is a multifaceted process encompassing numerous intricate mechanisms. Despite noteworthy advancements that have been achieved within this domain, the scope and depth of pertinent research remain limited. There is a pressing need for further exploration to broaden the understanding of this complex phenomenon and to deepen the insights gained from existing studies. Given the prevailing research focal points, we propose the adoption of the following strategies to propel the advancement of this field in a more precise and efficient trajectory: (1) using artificial intelligence technology to assist in the collation and analysis of data from patients with RHF ventricular remodeling; (2) constructing a large-scale, multicenter database of patients with RHF ventricular remodeling; and (3) combining the multi-homological data related to RHF ventricular remodeling to undertake a comprehensive study. The over-activation of neurohormones plays a central role in RHF ventricular remodeling. For example, abnormal secretion of hormones such as angiotensin and aldosterone will directly lead to the emergence of remodeling phenomena such as myocardial hypertrophy and interstitial fibrosis. However, due to the constraints and limitations of current research, evidence on the effectiveness of RAAS inhibition therapy in patient populations with right heart failure is still lacking in clinical practice. This emphasizes the urgency and importance of further in-depth research and validation of the efficacy of RAAS inhibition therapy in these patient populations. Activation of the SNS results in increased sensitivity of cardiomyocytes to CA, further promoting apoptosis and necrosis of cardiomyocytes. Under oxidative stress, the production of reactive oxygen radicals in cardiomyocytes increases, leading to cardiomyocyte injury and apoptosis and promotes the development of ventricular remodeling. Mechanisms such as aberrant expression of cytokines, remodeling of the ECM, and apoptosis of cardiomyocytes are interrelated and interact with each other, and together, they participate in the complex process of RHF ventricular remodeling. Therefore, therapeutic approaches targeting these mechanisms are important for improving the symptoms of RHF. By modulating neurohormone secretion, inhibiting SNS activation, attenuating oxidative stress, regulating cytokine expression, and improving ECM remodeling, the process of ventricular remodeling can be effectively decreased, thereby improving the prognosis and quality of life of patients with RHF. Future studies should further explore the specific details of these mechanisms to provide more effective strategies and methods for treating RHF.
